# Elevated Pressure Changes the Purinergic System of Microglial Cells

**DOI:** 10.3389/fphar.2018.00016

**Published:** 2018-01-24

**Authors:** Ana C. Rodrigues-Neves, Inês D. Aires, Joana Vindeirinho, Raquel Boia, Maria H. Madeira, Francisco Q. Gonçalves, Rodrigo A. Cunha, Paulo F. Santos, António F. Ambrósio, Ana R. Santiago

**Affiliations:** ^1^Coimbra Institute for Clinical and Biomedical Research (iCBR), Faculty of Medicine, University of Coimbra, Coimbra, Portugal; ^2^CNC.IBILI Consortium, University of Coimbra, Coimbra, Portugal; ^3^Center for Neuroscience and Cell Biology, University of Coimbra, Coimbra, Portugal; ^4^Faculty of Medicine, University of Coimbra, Coimbra, Portugal; ^5^Department of Life Sciences, Faculty of Sciences and Technology, University of Coimbra, Coimbra, Portugal; ^6^Association for Innovation and Biomedical Research on Light and Image, Coimbra, Portugal

**Keywords:** purinergic system, ATP, adenosine, microglia, elevated pressure, glaucoma

## Abstract

Glaucoma is the second cause of blindness worldwide and is characterized by the degeneration of retinal ganglion cells (RGCs) and optic nerve atrophy. Increased microglia reactivity is an early event in glaucoma that may precede the loss of RGCs, suggesting that microglia and neuroinflammation are involved in the pathophysiology of this disease. Although global changes of the purinergic system have been reported in experimental and human glaucoma, it is not known if this is due to alterations of the purinergic system of microglial cells, the resident immune cells of the central nervous system. We now studied if elevated hydrostatic pressure (EHP), mimicking ocular hypertension, changed the extracellular levels of ATP and adenosine and the expression, density and activity of enzymes, transporters and receptors defining the purinergic system. The exposure of the murine microglial BV-2 cell line to EHP increased the extracellular levels of ATP and adenosine, increased the density of ecto-nucleoside triphosphate diphosphohydrolase 1 (E-NTPDase1, CD39) and decreased the density of the equilibrative nucleotide transporter 2 as well as the activity of adenosine deaminase. The expression of adenosine A_1_ receptor also decreased, but the adenosine A_3_ receptor was not affected. Notably, ATP and adenosine selectively control migration rather than phagocytosis, both bolstered by EHP. The results show that the purinergic system is altered in microglia in conditions of elevated pressure. Understanding the impact of elevated pressure on the purinergic system will help to unravel the mechanisms underlying inflammation and neurodegeneration associated with glaucoma.

## Introduction

Glaucoma is a multifactorial degenerative disease characterized by the progressive loss of retinal ganglion cells (RGCs) and optic nerve fibers that leads to vision loss (Casson et al., 2012). Elevated intraocular pressure (IOP) is the most common risk factor of glaucoma and the current treatments are based on lowering the IOP (Cohen and Pasquale, 2014). However, some patients still progress to blindness despite successful control of IOP (Song et al., 2015), suggesting that the control of IOP is not sufficient to prevent RGC loss (Levin et al., 2017).

Microglial cells are the immunocompetent cells of the central nervous system (CNS). Under resting conditions, microglial cells are ramified and constantly monitor their surrounding environment. However, after an insult, microglia change their morphology and become reactive, migrate toward the lesion site, release pro-inflammatory mediators and phagocyte cell debris (Ransohoff and Perry, 2009). Sustained microglia-mediated neuroinflammation is associated with several retinal neurodegenerative diseases, namely glaucoma (Madeira et al., 2015a). Exacerbated microglia reactivity has been detected early in experimental models of glaucoma, even before RGC death (Bosco et al., 2011, 2015), suggesting that the modulation of microglia reactivity may attenuate disease progression.

Purine nucleotides are, among others, primordial mediators of cell-to-cell communication (Burnstock and Verkhratsky, 2009; Verkhratsky and Burnstock, 2014). Particularly, purines control several functions of microglia, such as chemotaxis, phagocytosis, and cytokine and chemokine release (reviewed in Di Virgilio et al., 2009). In pathological conditions, neurons, astrocytes and microglia release ATP and adenosine to the extracellular space (Sperlágh and Illes, 2007; Rodrigues et al., 2015). The ratio between the release and the removal, by enzymatic degradation and uptake, determines the extracellular levels of ATP and adenosine. ATP has been postulated to be released by vesicular exocytosis, carrier-mediated release, cytolytic release and through channels and membrane pores (Ballerini et al., 2002; Cook and McCleskey, 2002; Stout et al., 2002; Sperlágh and Illes, 2007; Gajardo-Gomez et al., 2016). Adenosine is considered to be generated by the breakdown of ATP through the actions of NTPDase (CD39) and ecto-5′-nucleotidase (CD73) (Yegutkin, 2008; Cunha, 2016), but it can also be released on its own (Sperlágh and Illes, 2007). The extracellular levels of adenosine are regulated by equilibrative nucleoside transporters (ENTs), that mediate the bidirectional adenosine transport, or through concentrative transporters (CNTs), that mediate the influx of adenosine (Yegutkin, 2008; Melani et al., 2012). Moreover, adenosine can be metabolized into adenosine monophosphate (AMP) by adenosine kinase (ADK) or into inosine by adenosine deaminase (ADA).

There are indications that the purinergic system is altered in glaucoma, as heralded by the increased levels of ATP detected in the aqueous humor of patients with glaucoma (Zhang et al., 2007; Li et al., 2011) and in experimental models of the disease (Resta et al., 2007; Reigada et al., 2008). Since microglial cells have a role in the pathophysiology of glaucoma and the purinergic system in microglial cells has been demonstrated to play a critical role in controlling inflammation and immune response in several models of neurological diseases (reviewed in Harry, 2013), we now studied the impact of elevated pressure in the set-up of the purinergic system in microglia and also assessed the role of ATP and adenosine in mediating the effects of elevated pressure in microglia phagocytosis and motility.

## Materials and methods

### Microglial cell culture

The immortalized murine BV-2 microglia cell line was cultured in Roswell Park Memorial Institute (RPMI) medium, supplemented with 10% fetal bovine serum (FBS) and 1% of antibiotics (penicillin 100 U/ml, streptomycin 100 μg/ml) and maintained at 37°C under a humidified atmosphere with 5% CO_2_. For experiments, cells were cultured in RPMI with 2% FBS and 1% antibiotics at a density of 6 × 10^3^ cells/cm^2^ in 6-well-plates or at density of 1 × 10^4^ cells/cm^2^ in 12-well-plates.

Cells were exposed to elevated hydrostatic pressure (EHP, 70 mmHg above atmospheric pressure) for 4 or 24 h, as previously described (Madeira et al., 2016). Control cultures were kept in a standard incubator at atmospheric pressure.

### ATP quantification

The extracellular levels of ATP were measured with a luciferin-luciferase assay kit, as previously described (Cunha et al., 2000; Madeira et al., 2015b). Briefly, cell culture supernatants were collected and stored at −80°C until used. Cells were collected and protein concentration was determined by the bicinchoninic acid (BCA) method (Pierce Biotechnology). Supernatants were quickly defrosted and incubated with ATP assay mix (Sigma-Aldrich) in an opaque 96-well plate. ATP levels were measured using a VICTOR multilabel plate reader (Perkin Elmer). The ATP concentration in each sample was determined by interpolation with a standard curve obtained from an ATP stock solution and was normalized to the amount of protein. Results are presented as percentage of control.

### Adenosine quantification

The extracellular levels of adenosine were quantified using high-performance liquid chromatography (HPLC), as previously described (Vindeirinho et al., 2013). Briefly, cell supernatants were analyzed in Beckman-System Gold HPLC apparatus, with a computer controlled 126 Binary Pump Model using a 166 Variable UV detector (detection at 254 nm) and a Lichrospher 100 RP-18 (5 mm) column from Merck. An isocratic elution with 10 mM phosphate buffer (NaH_2_PO_4_; pH 6.0) and 14% methanol was performed with a flow rate of 1.5 ml/min. Adenosine was quantified by considering the retention time and absorption spectra, and then comparing with the standard curve. Results are presented as percentage of control.

### Measurement of AMP dephosphorylation

BV-2 microglial cells were washed with warm reaction buffer (in mM: 2 MgCl_2_, 125 NaCl, 1 KCl, 10 glucose, 10 HEPES; pH 7.4), and then incubated with 2 mM adenosine monophosphate (AMP; Sigma-Aldrich) for 40 min at 37°C. The medium was collected to quantify the free inorganic phosphate, as a measurement of AMP degradation with the Malachite Green Phosphate Assay kit, following the instructions provided by the manufacturer (Cayman Chemicals). Inorganic free phosphate was detected by spectrophotometry (620–630 nm). Results are presented as fold-change of control.

### Measurement of ADA activity

BV-2 cells were washed twice with ice-cold phosphate-buffered saline (PBS, in mM: 137 NaCl, 2.7 KCl, 10 Na_2_HPO_4_, 1.8 KH_2_PO_4_, pH 7.4) and lysed in 50 mM Tris-HCl (pH 7.2), supplemented with complete mini protease inhibitor cocktail tablets (Roche). The enzymatic activity of ADA was measured following the instructions provided by the manufacturer (Diazyme). Results were normalized to the amount of protein quantified by the BCA method, and are presented as percentage of control.

### Western blotting

BV-2 cells were washed twice with ice-cold PBS at 4°C. Cells were lysed in radioimmunoprecipitation assay buffer [RIPA, in mM: 50 Tris HCl, pH 7.4; 150 NaCl; 5 EDTA; 1% Triton X-100; 0.5% sodium deoxycholate; 0.1% sodium dodecyl sulfate (SDS)] supplemented with complete mini protease inhibitor cocktail tablets and 1 mM of dithiothreitol (DTT) at 4°C. Samples were denatured by adding 6x concentrated sample buffer (0.5 M Tris, 30% glycerol, 10% SDS, 0.6 M DTT, 0.012% bromophenol blue) and heating for 5 min at 95°C. When blotting for CD39, cells were lysed in non-reduced conditions.

Samples were separated in SDS-polyacrylamide gel electrophoresis (SDS-PAGE) and transferred onto PVDF membranes (Millipore). The membranes were blocked with 5% milk and then were incubated with the antibodies indicated in Table 1. Immunoreactive bands were visualized using Enhanced Chemi-Fluorescence system (ECF; GE Healthcare) on a Storm device (Molecular Dynamics, GE Healthcare) or with Enhance Chemiluminescence system (ECL; Bio-Rad) on a Versadoc (Bio-Rad). Digital quantification of band intensity was performed using Quantity One software (Bio-Rad). The membranes were reprobed for glyceraldehyde 3-phosphate dehydrogenase (GAPDH) to control for similar amounts of protein.

**Table 1:** List of primary antibodies used in Western blotting.

**Primary antibody**	**Dilution**	**Protein (μg)**	**Molecular weight (kDa)**	**Supplier**
Anti-A_1_R	1:500	30	37	Thermo Fisher Scientific
Anti-A_3_R	1:100	60	44	Santa Cruz Biotechnology
Anti-ADA	1:100	60	41	Santa Cruz Biotechnology
Anti-ADK	1:100	60	48/38	Santa Cruz Biotechnology
Anti-CNT2	1:100	60	72	Santa Cruz Biotechnology
Anti-CD39	1:100	70	~70	Ectonucleotidases-ab
Anti-ENT2	1:100	40	50-55	Santa Cruz Biotechnology
Anti-GAPDH	1:5000	–	37	Abcam

### Immunocytochemistry

Cells were washed with ice-cold PBS and fixed using 4% of paraformaldehyde (PFA) with 4% sucrose for 10 min at room temperature (RT). After fixation, the cells were washed with PBS and permeabilized during 5 min with 1% Triton X-100 in PBS. Then, cells were blocked with 3% of bovine serum albumin (BSA; NZYtech) and 0.2% Tween-20 in PBS for 1 h at RT to prevent the non-specific binding. Afterwards, cells were incubated with the primary antibody diluted in the blocking solution for 90 min at RT (Table 2). After washing, the cells were incubated with the secondary antibody in the same solution for 1 h at RT in the dark. Then, cells were rinsed with PBS. Nuclei were stained with 4′,6-diamidino-2-phenylindole (DAPI 1:2,000; Molecular Probes, Life Technologies) and F-actin was stained with phalloidin conjugated to Tetramethylrhodamine B isothiocyanate (TRITC, 1:500; Sigma-Aldrich). Upon rising with PBS, the coverslips were mounted in glycergel mounting medium and observed with a fluorescence microscope (Axio Observer.Z1), using a LD Plan-Neofluar 40x/0.6 Korr Ph2 M27 objective.

**Table 2:** List of primary antibodies used in immunocytochemistry.

**Primary antibody**	**Dilution**	**Supplier**
A_1_R	1:250	Thermo Fisher Scientific
A_3_R	1:50	Santa Cruz Biotechnology

Representative images were acquired with a confocal microscope (LSM 710, Zeiss) on an Axio Observer.Z1 microscope using Plan-Apochromat 63x/1.40 Oil Dic M27 objective.

### Boyden chamber migration assay

BV-2 cells were cultured in serum-free medium for 24 h before the experiment. Then, cells were plated in transwell cell culture inserts (8.0 μm pore diameter; Merck Millipore) in RPMI with 2% FBS and 1% antibiotics. Cells were incubated with apyrase (30 U/mL) and ADA (1 U/mL) followed by exposure to EHP for 4 h. At the end of the experiment, cells were washed with warm PBS and the cells in upper side of the insert were removed with a cotton swab. After fixation with 4% PFA with 4% sucrose during 10 min, the nuclei were stained with DAPI (1:2,000). The membranes were mounted in glass slides with glycergel mounting medium, and the preparations were observed with a fluorescence microscope (Axio Observer.Z1) using a N-Achroplan 5x/0.15 M27 objective. Five random images per sample were acquired. The number of cells in the bottom side of the insert (the cells that migrated) was counted and the results were expressed as percentage of control.

### Phagocytosis assay

BV-2 cells were plated in RPMI with 2% FBS and 1% antibiotics and exposed to EHP for 24 h. Cells were incubated with apyrase (30 U/mL) and ADA (1 U/mL) 2 h before the end of the experiment. One hour before the end of the experiment, BV-2 cells were incubated with 0.025% fluorescent beads at 37°C. Then, cells were washed with warm PBS and fixed with 4% PFA with 4% sucrose. BV-2 cells were stained with TRITC-conjugated phalloidin (1:500; Sigma-Aldrich) and nuclei were stained with DAPI (1:2,000). Cells were observed with a confocal microscope (LSM 710, Zeiss) using a Plan-Apochromat 20x/0.8 M27 objective and five random fields were acquired from each condition. The total number of cells in each field, the number of cells with incorporated beads and the number of fluorescent beads phagocytized by each cell were counted. The phagocytic efficiency was calculated:

$$\begin{array}{l} Phagocytic\;efficiency\;\left(\%\right)=\frac{\left(1\;\times \;x1+2\;\times \;x2+3\;\times \;x3+n\;\times \;xn\right)}{total\;number\;of\;cells}\\ \;\times \;100\% \end{array}$$

where *xn* represents the number of cells containing *n* microspheres (*n* = 1, 2, 3 … up to a maximum of 6 points for more than 5 microspheres ingested per each cell).

### Statistical analysis

Results are presented as mean ± SEM. The number of independent experiments is indicated in each bar. Statistical analysis was performed using GraphPad Prism Version 6 (GraphPad Software). The normality of the data was assessed with Shapiro-Wilk test. Data was analyzed using the non-parametric Kruskall-Wallis test, followed by Dunn's multiple comparison test. Differences were considered significant for *p* < 0.05.

## Results

Microglial cells are endowed with the machinery of the purinergic system (Sperlágh and Illes, 2007; Castellano et al., 2016). We now assessed how the purinergic system of microglial cells is altered after challenging the microglial cells in a pressure chamber to mimic elevated IOP.

### Elevated hydrostatic pressure increases extracellular levels of ATP and adenosine

**Figure 1:**
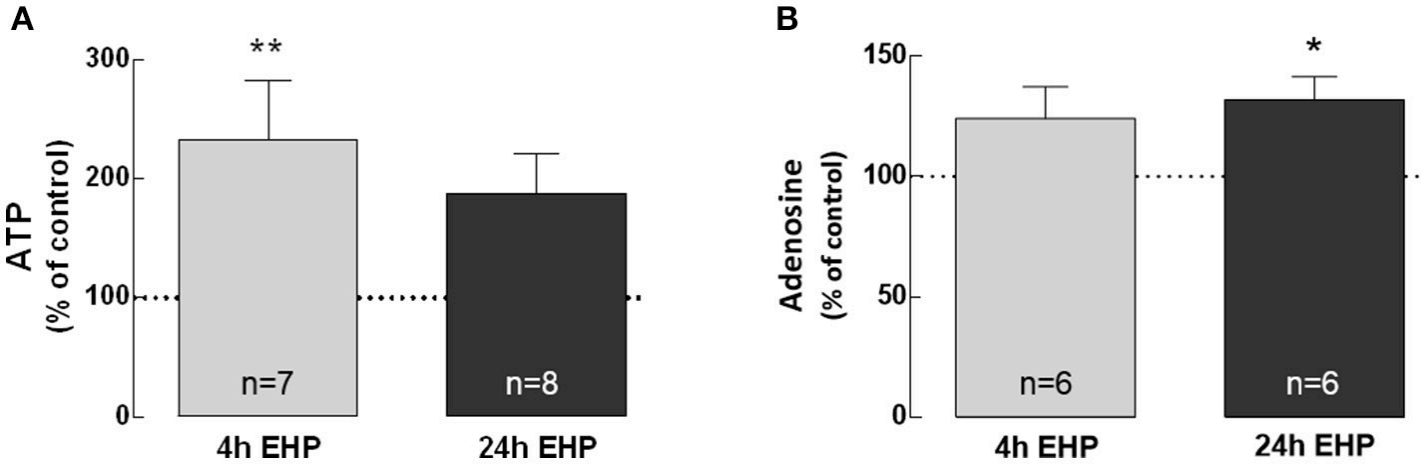
Elevated hydrostatic pressure increases extracellular levels of ATP and adenosine. The levels of extracellular ATP **(A)** and adenosine **(B)** were quantified in cell supernatants. Results were normalized to the amount of protein in each sample and are expressed as percentage of the control. ^*^p < 0.05, ^**^p < 0.01, different from control; Kruskal-Wallis test, followed by Dunn's multiple comparison test.

BV-2 cells were exposed to elevated pressure for 4 and 24 h and the levels of ATP (Figure 1A) and adenosine (Figure 1B) were quantified in cell culture medium supernatants. The exposure of microglia to EHP for 4 and 24 h increased the extracellular levels of ATP to 233.1 ± 49.9% (*p* < 0.01) and 187.9 ± 33.4% of control, respectively, and the adenosine levels to 124.1 ± 9.6% and 131.9 ± 9.6% of the control (*p* < 0.05), respectively.

### Elevated hydrostatic pressure increases CD39 but does not affect AMP catabolism

Adenosine can be formed through the hydrolysis of adenine nucleotides [ATP, adenosine di-phosphate (ADP) and AMP] by a cascade of ectonucleotidases, including CD39 and CD73 that are expressed in several cell types, including microglia (Haskó et al., 2005).

**Figure 2:**
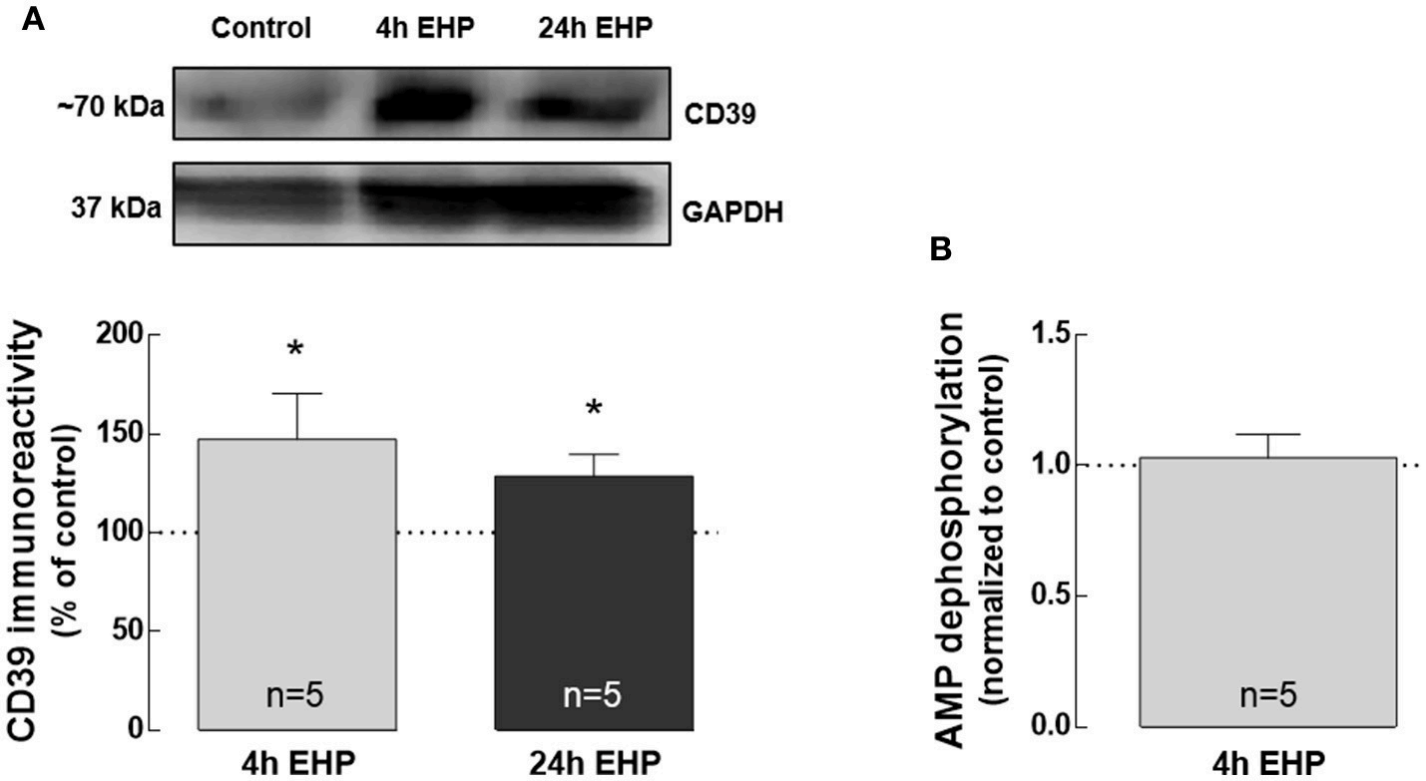
Elevated hydrostatic pressure increases CD39 but does not affect AMP catabolism. Total BV-2 cell extracts were assayed for CD39 **(A)** by Western blot. Representative images for CD39 and GAPDH (loading control) are presented above the graph. Results are expressed as percentage of control. **(B)** AMP dephosphorylation was evaluated by the malachite green phosphate assay in cell supernatants. Results are expressed as fold change of control. ^*^p < 0.05, different from control; Kruskal-Wallis test, followed by Dunn's multiple comparison test.

Here, we addressed whether EHP could affect the expression of CD39 as well as AMP catabolism, both involved in adenosine formation through ATP hydrolysis. CD73 was not detected in BV-2 cells either by qPCR or Western blot (data not shown). The protein levels of CD39 significantly increased in BV-2 cells exposed to EHP for 4 and 24 h (147.3 ± 23.1% and 128.6 ± 11.0% of the control, respectively; *p* < 0.05; Figure 2A), which is in agreement with the previous proposal that CD39 might be a potential indicator of increased extracellular levels of ATP in retina cells (Lu et al., 2007). However, the dephosphorylation of AMP into adenosine was not altered in BV-2 cells exposed to 4 h EHP (1.0 ± 1 fold-change; Figure 2B).

### Elevated hydrostatic pressure impairs the activity of ADA, but not the protein levels of ADA and ADK

Adenosine can be removed from the extracellular space by degradation into inosine mediated by ADA, while the intracellular the removal of adenosine also occurs by the conversion into AMP mediated by adenosine kinase (Yegutkin, 2008).

**Figure 3:**
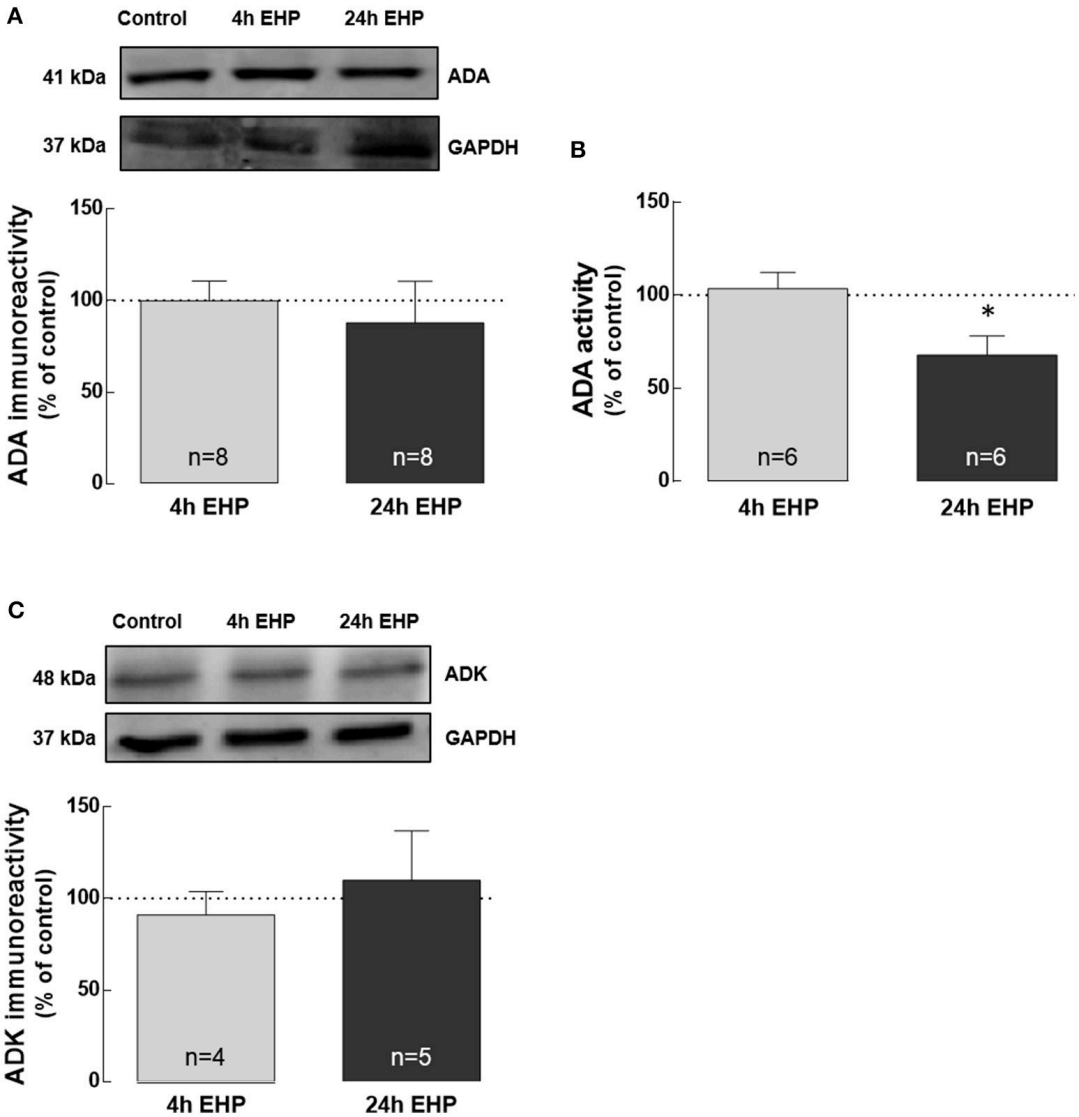
Elevated hydrostatic pressure impairs the activity of ADA, but not the protein levels of ADA and ADK. Total BV-2 cell extracts were assayed for ADA by Western blot **(A)** and enzymatic activity **(B)** as well as for ADK by Western blot **(C)**. Representative images for ADA, ADK and GAPDH (loading control) are presented above the graphs **(A**,**C)**. Results are expressed as percentage of control. ^*^p < 0.05, different from control; Kruskal-Wallis test, followed by Dunn's multiple comparison test.

The exposure of microglial cells to EHP for 4 and 24 h did not affect the protein levels of ADA and ADK (Figures 3A,C). Nevertheless, the activity of ADA (Figure 3B) significantly decreased in microglia exposed to EHP for 24 h to 67.9 ± 10.2% of the control (*p* < 0.05).

### Elevated hydrostatic pressure decreases ENT2 but does not change CNT2 protein levels

Extracellular adenosine levels are typically regulated by nucleoside transporters or metabolism (Latini and Pedata, 2001). Hence, we also evaluated the impact of EHP exposure on the expression of nucleoside transporters ENT1, ENT2, and CNT2 in BV-2 cells.

**Figure 4:**
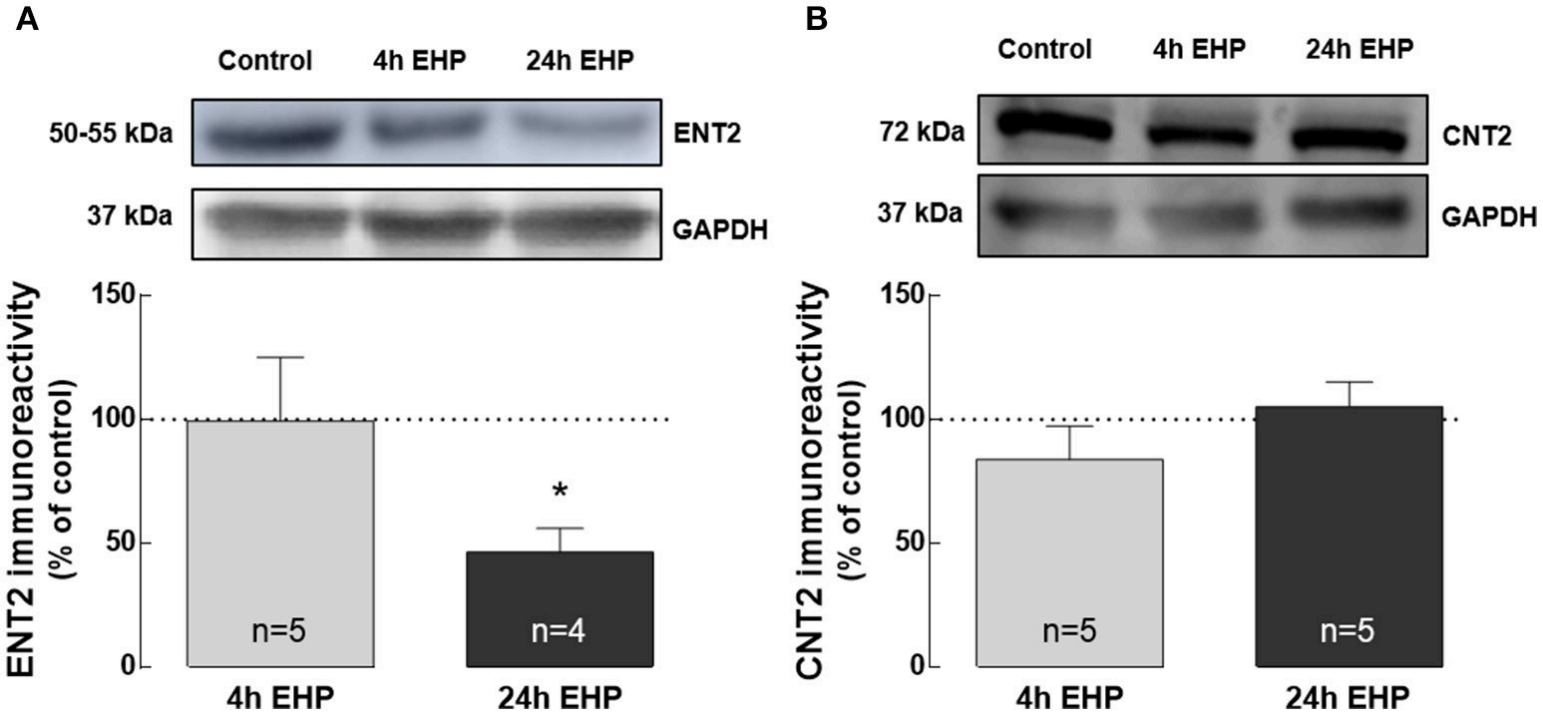
Elevated hydrostatic pressure decreases ENT2 but does not change CNT2 protein levels. Total BV-2 cell extracts were assayed for ENT2 **(A)** and CNT2 **(B)** by Western blot. Representative images for ENT2, CNT2, and GAPDH (loading control) are presented above the graphs. Results are expressed as percentage of control. ^*^p < 0.05, different from control; Kruskal-Wallis test, followed by Dunn's multiple comparison test.

ENT1 was not detected by Western blot (data not shown). The protein levels of ENT2 in BV-2 microglia exposed to EHP for 4 h were similar to the control. However, exposure to EHP for 24 h significantly decreased the protein levels of ENT2 in microglia (46.5 ± 9.6% of the control; *p* < 0.05; Figure 4A). The protein levels of CNT2 were not altered in BV-2 cells exposed to EHP (83.8 ± 13.5% and 105.2 ± 10.0% of the control, for 4 and 24 h, respectively; Figure 4B).

### Elevated hydrostatic pressure changes the expression and density of A_1_R but not A_3_R

The actions of adenosine are dependent on the activation of the adenosine receptors. We evaluated the impact of EHP on the expression of A_1_R and A_3_R receptors in BV-2 microglial cells.

**Figure 5:**
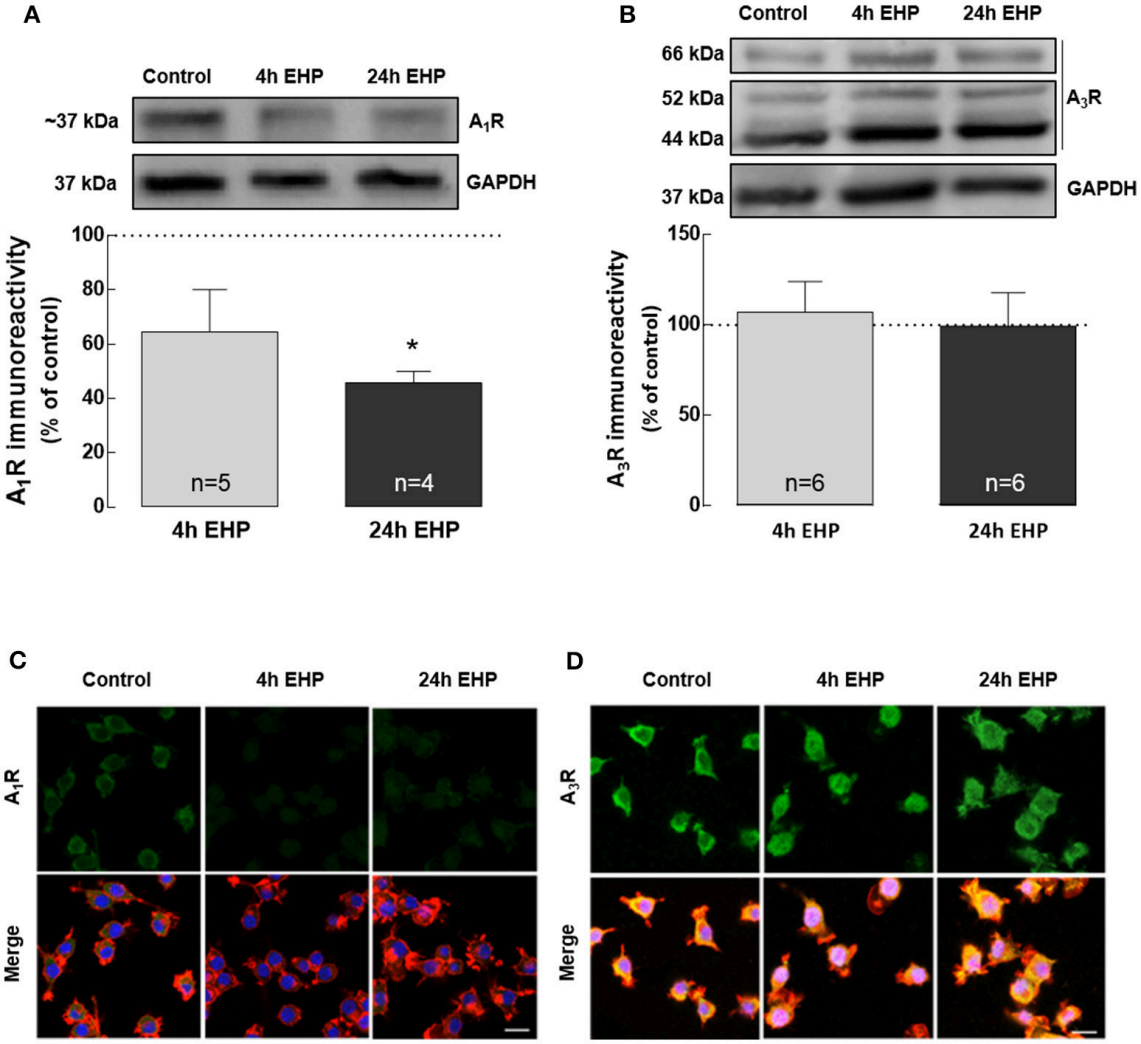
Elevated hydrostatic pressure changes the expression and density of A_1_R but not A_3_R. Total BV-2 cell extracts were assayed for A_1_R **(A)** and A_3_R **(B)** by Western blot. Representative images for A_1_R, A_3_R and GAPDH (loading control) are presented above the graphs. Results are expressed as percentage of control. Cells were immunolabelled with anti-A_1_R (green) **(C)** or anti-A_3_R (green) **(D)** antibodies. Phalloidin (red) staining was used to visualize cells. Nuclei were counterstained with DAPI (blue). Scale bar: 20 μm. ^*^p < 0.05, different from control; Kruskal-Wallis test, followed by Dunn's multiple comparison test.

The exposure of BV-2 cells to EHP, for 4 and 24 h, decreased the protein levels of A_1_R to 64.5 ± 15.6% and 45.9 ± 4.2% (*p* < 0.05) of the control, respectively, as assessed by Western blot (Figure 5A). As we previously reported (Lopes et al., 2003), Western blot for A_3_R yields three bands but we quantified the more intense 44 kDa that corresponds to the expected molecular weight of A_3_R. This analysis revealed that the protein levels of A_3_R (Figure 5B) did not change in microglial cells upon exposure to EHP for 4 and 24 h (97.4 ± 17.2% and 124.0 ± 27.8% of the control, respectively).

The effects of EHP on the immunoreactivity of A_1_R and A_3_R were also assessed by immunocytochemistry in microglia (Figures 5C,D). In microglial cells exposed to EHP, A_1_R immunoreactivity decreased when compared with the control, whereas the A_3_R immunoreactivity was similar to the control, supporting the results obtained by Western blotting.

### ATP and adenosine mediate microglia migration induced by elevated hydrostatic pressure

**Figure 6:**
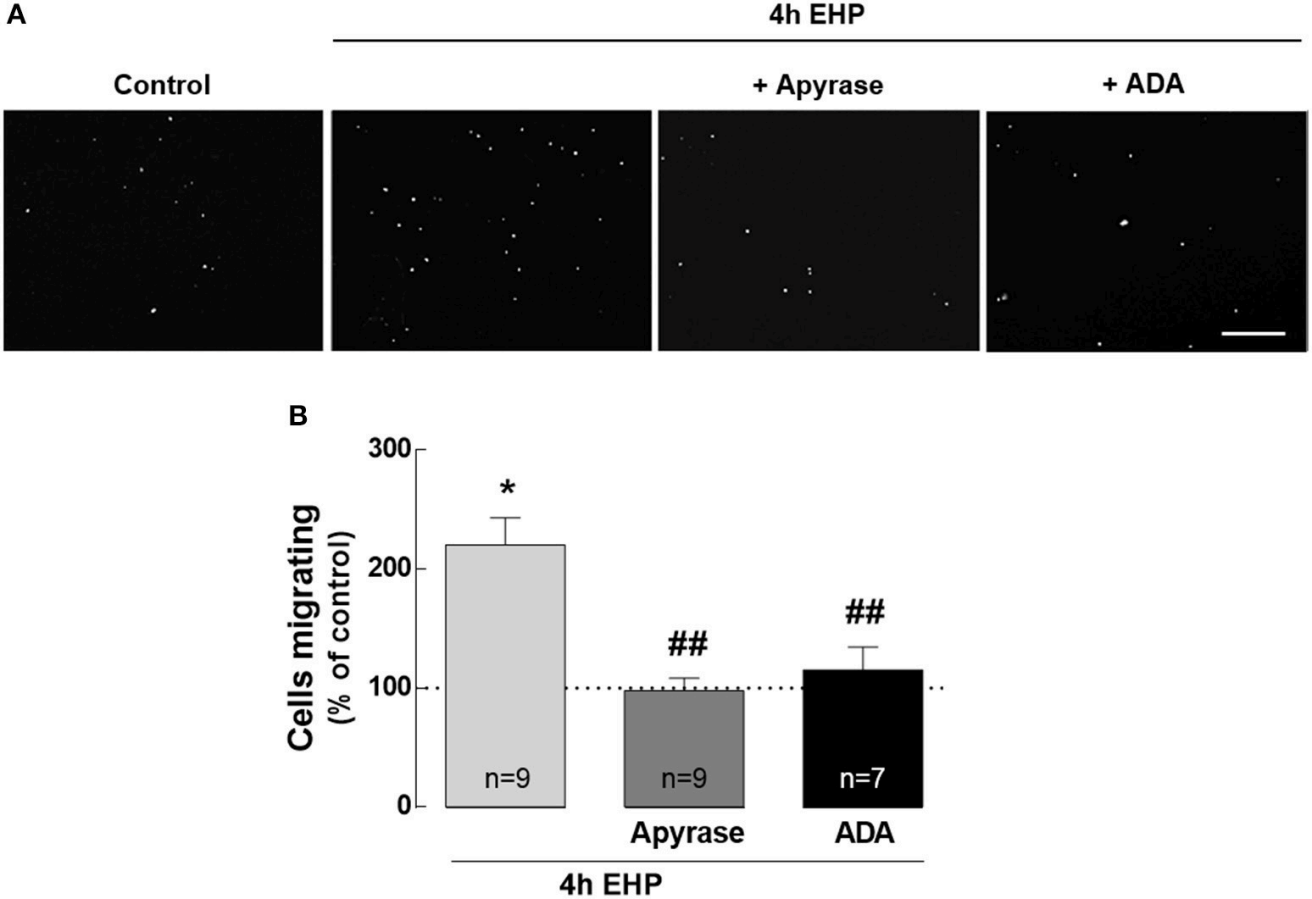
ATP and adenosine mediate microglia migration induced by elevated hydrostatic pressure. Cell migration was assessed with the Boyden chamber migration assay. The cell nuclei in the bottom surface of the Transwell were observed after DAPI staining **(A)** and the number of migrated BV-2 cells was counted. **(B)** Results are expressed as percentage of control. Scale bar: 100 μm. ^*^p < 0.05, different from control; ^##^*p* < 0.01, different from 4 h EHP; Kruskal-Wallis test, followed by Dunn's multiple comparison test.

A feature of reactive microglia is their ability to migrate toward the site of injury, where they release pro-inflammatory mediators and phagocyte cell debris (Karlstetter et al., 2010). Although the role of the purinergic system in microglia chemotaxis is well established (Honda et al., 2001; Davalos et al., 2005; Wu et al., 2007), the contribution of ATP and adenosine to the motility of microglia in conditions of elevated pressure it still unknown. Therefore, we evaluated whether EHP could affect microglia migration and if ATP and adenosine affected this process (Figure 6). When microglial cells were exposed to EHP for 4 h, the number of microglia that migrated to the bottom side of the transwell significantly increased to 220.5 ± 22.8% of the control (*p* < 0.05). The treatment of BV-2 cells with 30 U/ml apyrase, to remove the extracellular ATP, prevented the effect of EHP in microglia migration (98.0 ± 10.4% of the control; *p* < 0.01 compared with EHP). BV-2 cells were also pre-treated with 1 U/ml ADA, to remove the extracellular adenosine. The removal of extracellular adenosine also prevented the migration of microglial cells to the bottom side of the membrane elicited by EHP (115.5 ± 19.2% of the control, *p* < 0.01 compared with EHP). This strict dependency on extracellular ATP and adenosine for the increased migration of microglia upon EHP is also an important control to exclude the possibility that migration in EHP conditions might result from cells that were just pushed through the pores.

### ATP and adenosine do not contribute to the increase of phagocytic efficiency in microglia induced by elevated hydrostatic pressure

**Figure 7:**
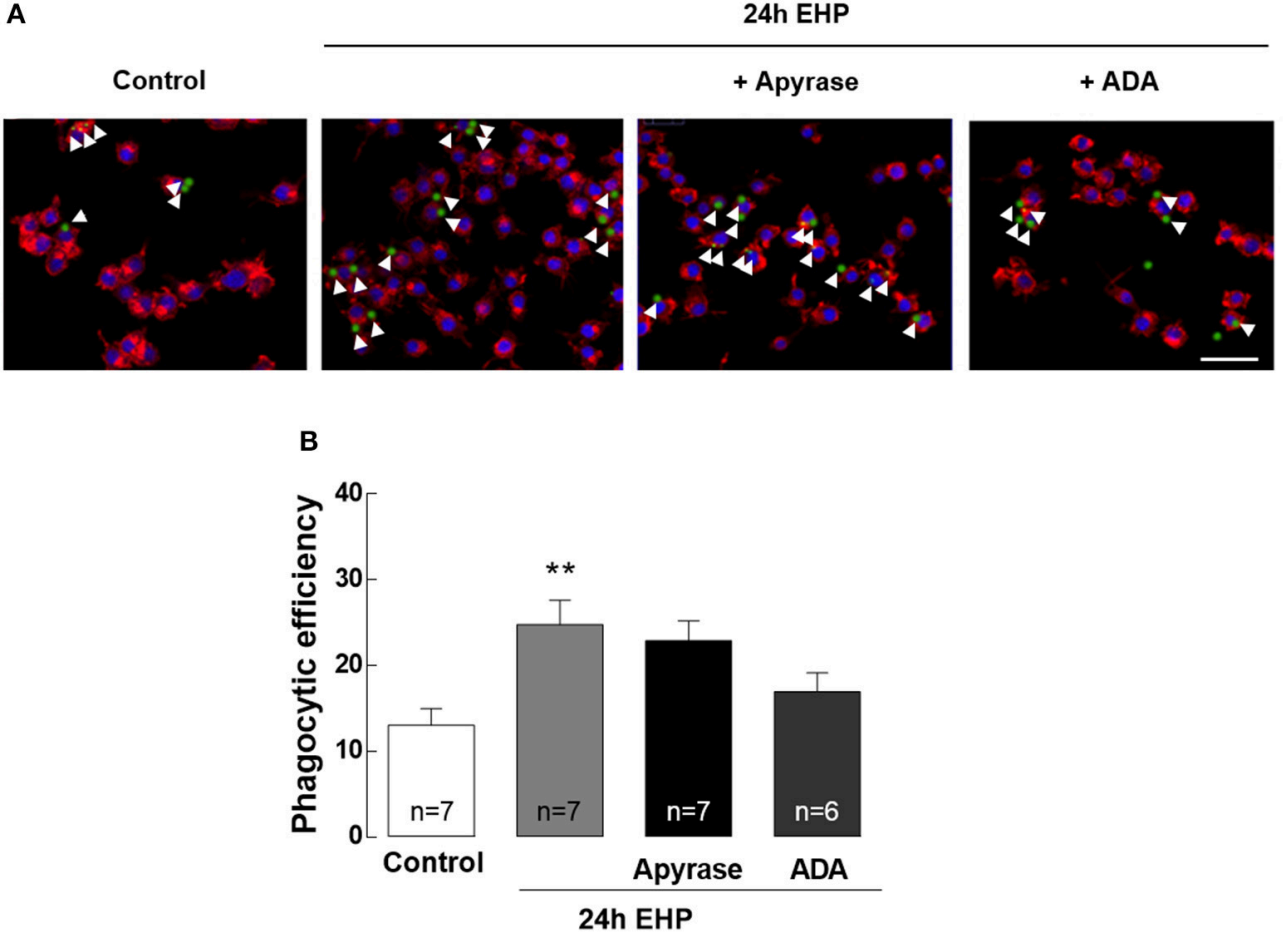
ATP and adenosine do not contribute to the increase of phagocytic efficiency in microglia induced by elevated hydrostatic pressure. Phagocytosis was assessed using fluorescent microbeads. **(A)** Representative images of BV-2 cells stained with phalloidin (red) with incorporated beads (green). Nuclei were counterstained with DAPI (blue). Arrowheads show some beads engulfed by microglia. **(B)** Phagocytic efficiency. Scale bar: 50 μm. ^**^p < 0.01, different from control; Kruskal-Wallis test, followed by Dunn's multiple comparison test.

Increased phagocytic efficiency is a feature of reactive microglia (Kettenmann et al., 2011). The contribution of adenosine and ATP to the phagocytic efficiency of microglia upon exposure to EHP was assessed by incubating microglia with fluorescent microbeads (Figure 7). The phagocytic efficiency of microglia exposed to EHP increased by nearly 200% when compared with control cells (from 13 ± 2% in control conditions to 25 ± 3% under EHP; *p* < 0.01). Incubation with apyrase (30 U/ml) and ADA (1 U/ml) was unable to decrease the phagocytic efficiency in cells under EHP (23 ± 2% and 19 ± 3%, respectively).

## Discussion

The purinergic system is a controller of inflammation (reviewed in Di Virgilio et al., 2009) and microglia-mediated neuroinflammation contributes to the pathogenesis of glaucoma (reviewed in Madeira et al., 2015a). Here, we show for the first time that elevated pressure modifies the purinergic system of BV-2 microglial cells, altering the levels of ATP and adenosine, as well as enzymes responsible for the maintenance of purine levels, which we showed to regulate the EHP-induced microglial cell migration.

EHP increased the extracellular levels of ATP in BV-2 cells, without increasing cell death (data not shown), indicating that ATP released from these cells occurs by a non-lytic mechanism. Several reports have demonstrated the ability of microglia to release ATP under different conditions (Ferrari et al., 1997; Imura et al., 2013; Shinozaki et al., 2014; George et al., 2015). Indeed, microglia are endowed with the nucleotide transporter (VNUT) that transports cytosolic ATP into vesicles (Sawada et al., 2008) and the genetic deletion of VNUT impairs ATP release from microglia (Shinozaki et al., 2014). Other mechanisms mediate the release of ATP in microglia, namely, the carrier-mediated release (Ballerini et al., 2002), pannexins and/or hemi-channels (Gajardo-Gomez et al., 2016), as well as the cytolytic release (Sperlágh and Illes, 2007).

Increased ATP and adenosine levels have been reported in the aqueous humor of glaucoma patients (Daines et al., 2003; Zhang et al., 2007; Li et al., 2011), implicating the purinergic system in the pathophysiology of glaucoma. Increased ATP levels have been also documented in the retinas of animal models of glaucoma (Lu et al., 2015; Pérez de Lara et al., 2015) and in the vitreous of bovine eyecup preparations subjected to elevated pressure (Reigada et al., 2008). Moreover, we have recently reported that elevated pressure increases extracellular ATP in rat retinal organotypic cultures (Madeira et al., 2015b). In the retinal eyecup and organotypic models it is not possible to identify which cells release ATP, even though several cells are potential sources of extracellular ATP, such as neurons, astrocytes, Müller cells, and microglia (Rodrigues et al., 2015). Astrocytes, for instance, can release ATP when subjected to chronic mechanical strain (Beckel et al., 2014). Under mechanical strain, NLRP3 inflammasome is activated in the retina and astrocytes, due to the upregulation of IL-1β expression, which depends on ATP (released from pannexin hemichannels) and P_2_X_7_ receptor stimulation (Albalawi et al., 2017).

The levels of extracellular adenosine were increased in BV-2 microglia exposed to elevated pressure. Extracellular adenosine can arise from the direct release of adenosine into the extracellular space, or by ATP hydrolysis through ecto-enzymes (Yegutkin, 2008; Cunha, 2016), involving the conversion of ATP or ADP into AMP by CD39, followed by AMP dephosphorylation into adenosine by ecto-5′-nucleotidase (Ecto5′NTase, CD73). Increased expression of NTPDase1 in the posterior eye of rat, mouse, and primate models of glaucoma has been demonstrated (Lu et al., 2015), which is in line with our findings of increased CD39 protein levels in BV-2 cells under elevated pressure. Indeed, NTPDase1 was already proposed as an index for increased ATP levels under pathological conditions (Lu et al., 2007). However, AMP dephosphorylation in microglial cells was not altered by elevated pressure. In addition, we were unable to detect CD73, which is in accordance with a recent study that reported the absence of CD73 expression in the BV-2 cell line (Murphy et al., 2017). Importantly, other enzymes, such as alkaline phosphatase, widely distributed in cell surface, can also degrade ATP, ADP, and AMP into adenosine (Zimmermann, 2006; Yegutkin, 2008; Vardy et al., 2012), and may be responsible for the conversion of extracellular AMP into adenosine in these cells. The half-life of extracellular adenosine may be regulated by extracellular adenosine deaminase (Ecto-ADA) that irreversibly deaminates adenosine into inosine (Regateiro et al., 2013). Although no alterations were detected in the protein levels of ADA, the activity of this enzyme was decreased in cells exposed to elevated pressure, probably as a result of an allosteric modulation of ADA (Levine and Ginsburg, 2013), which we did not experimentally confirmed. This may explain the increase in extracellular adenosine levels. No changes were found in the protein levels of ADK (which converts intracellularly adenosine into AMP). Notably, this enzyme becomes easily saturated with basal concentrations of adenosine (Dunwiddie and Masino, 2001; Latini and Pedata, 2001), which prompts hypothesizing that ADK mostly controls basal levels of adenosine, but does not contribute to these increased levels of adenosine during elevated pressure.

Apart from metabolism, adenosine can also be cleared from the extracellular space by nucleoside transporters (Latini and Pedata, 2001). There are two types of adenosine transporters: equilibrative nucleoside transporters (ENTs) 1-4 and concentrative nucleoside transporters (CNTs) 1–3. Adenosine follows a concentrative gradient through ENTs, while CNTs transport nucleosides against the gradient (Thorn and Jarvis, 1996). ENTs are blocked by S-(4-nitrobenzyl)-6-thioinosine (NBMPR). In EOC-20 murine microglial cell line and in RAW264.7 macrophage cells, the majority of the adenosine transport is NBMPR-sensitive and insensitive to sodium removal, suggesting that ENTs are the primary transporters functioning in microglia (Carrier et al., 2006), where the presence of ENTs other than 1 and 2 has never been described. The decrease of ENT2 density in microglia caused by exposure to elevated pressure may affect the removal of adenosine from the extracellular space.

Adenosine mediates its actions by means of activation of G protein-coupled receptors named A_1_R, A_2A_R, A_2B_R, and A_3_R (Fredholm et al., 2001). All adenosine receptors have been described in microglial cells and microglia cell lines (Hammarberg et al., 2003; van Calker and Biber, 2005; Dare et al., 2007; Beckel et al., 2016). It is well documented that under brain noxious conditions, A_1_Rs are downregulated with a concomitant increase in A_2A_R density (reviewed in Cunha, 2005). Accordingly, we found that EHP caused A_1_R down-regulation in microglia and in previous reports, we demonstrated the up-regulation of A_2A_R in microglial cells triggered by elevated pressure (Madeira et al., 2015b, 2016). The downregulation of A_1_R may result from the exposure of the receptor to an excessive amount of adenosine that results in ligand-induced internalization of the receptor (Ciruela et al., 1997; Coelho et al., 2006).

The purinergic system regulates several functions of microglial cells, such as cell process extension and retraction, migration, proliferation, and phagocytosis (Davalos et al., 2005; Koizumi et al., 2007; Gomes et al., 2013; Matyash et al., 2017). Microglia migration increased under elevated pressure, and this process appears to be mediated by ATP and adenosine. Cell migration can occur by three mechanisms: basal motility (in the absence of a chemical stimulus), chemokinesis (random motility in response to a chemical stimulus), and chemotaxis (migration toward and dependent of a chemical gradient; Wilkinson, 1998; Miller and Stella, 2009). ATP is a known microglial chemotactic agent (Honda et al., 2001; Davalos et al., 2005; Wu et al., 2007) acting on P_2_Y_12_ and P_2_X_4_ receptors (Honda et al., 2001; Haynes et al., 2006). In addition to chemotaxis, ATP can mediate migration by chemokinesis (Miller and Stella, 2009). Moreover, adenosine may also play a role although it stills remains to be determined which adenosine receptor might bolster microglia chemotaxis: in fact A_2A_Rs are responsible for microglia retraction (Orr et al., 2009), whereas it was proposed that the activation of A_3_R is required for ADP-induced P_2_Y_12_-mediated migration of microglia (Ohsawa et al., 2012).

Phagocytosis is a crucial function of microglia both in the surveillance and in reactive states (Mandrekar et al., 2009; Karlstetter et al., 2014; Scheiblich and Bicker, 2016). Elevated pressure increased microglia phagocytosis, but extracellular ATP and adenosine failed to prevent the increased phagocytosis, indicating that ATP and adenosine do not control this microglial function under elevated pressure conditions. In addition, ATP and adenosine *per se* did not change the phagocytic efficiency under basal conditions (data not shown). Others reported that the dual activation of P1 and P2 are mandatory for both migration and phagocytic capacity of microglial cells (Bulavina et al., 2013), which is not in total accordance with our results. Therefore, we cannot discard the possibility that the synergistic action of ATP and adenosine could be pivotal in phagocytosis, during elevated pressure conditions.

**Figure 8:**
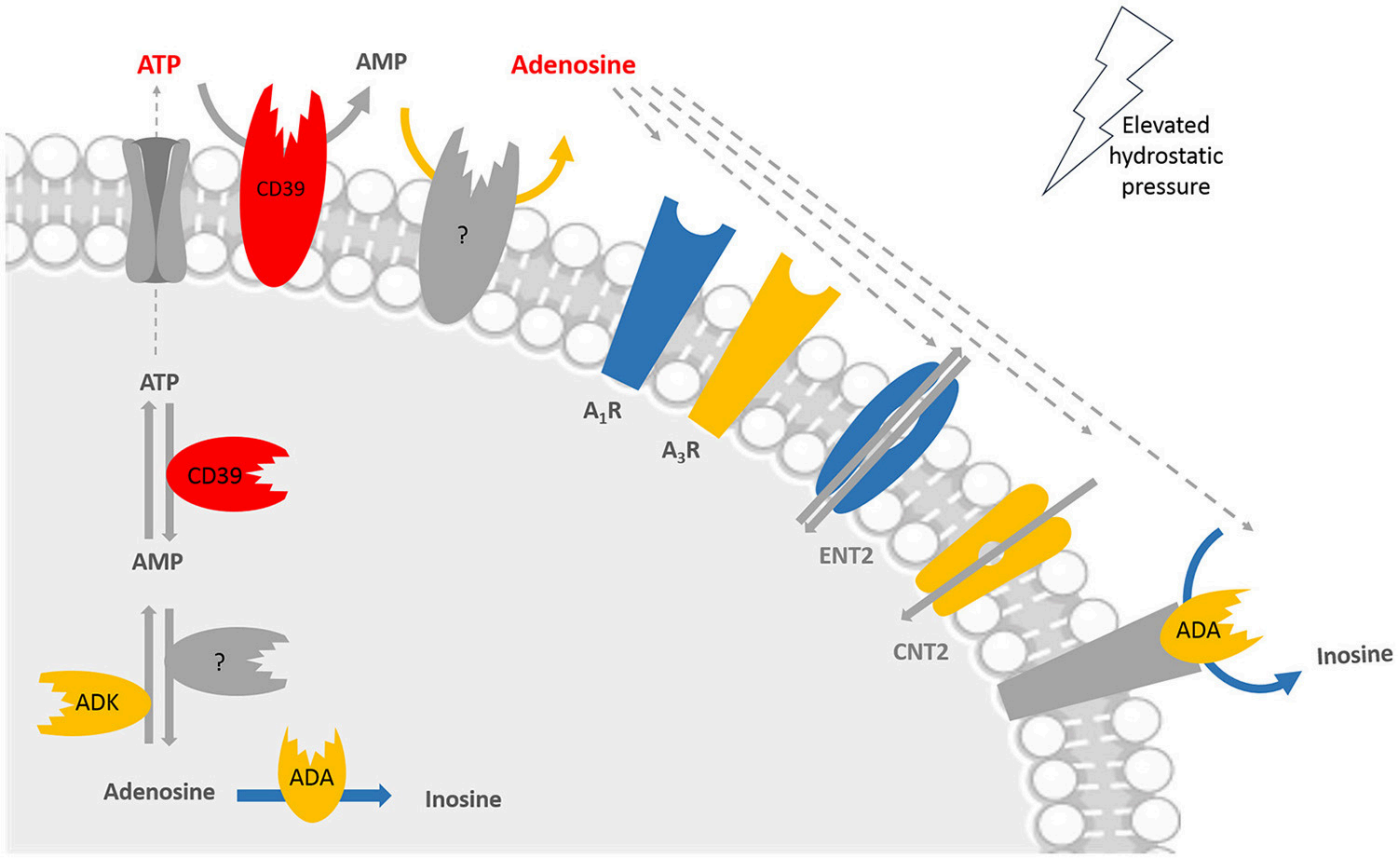
Main alterations in the purinergic system of microglial cells in conditions of elevated pressure. The exposure of microglial cells to elevated pressure changes several players of the purinergic system in microglia, including enzymes, receptors, and transporters. Color code: red means increased levels, expression, or density; blue means decreased expression, density, or activity; and yellow means no changes at elevated pressure when compared with control pressure. Taking into account the lack of CD73 in BV-2 cells, the enzyme responsible for converting AMP into adenosine was not identified; it could be for instance alkaline phosphatase (AP), but we did not assess it experimentally. Adenosine triphosphate (ATP), adenosine monophosphate (AMP), adenosine deaminase (ADA), adenosine kinase (ADK), adenosine A_1_ receptor (A_1_R), adenosine A_3_ receptor (A_3_R), concentrative nucleoside transporter 2 (CNT2), equilibrative nucleoside transporter 2 (ENT2).

Our data showed for the first time that elevated pressure impaired the purinergic system of microglial cells (Figure 8) and this could interfere with microglial functions under elevated pressure. While it is important to keep in mind that an *in vitro* model of elevated pressure is an over-simplified model of glaucoma and that the BV-2 microglia cell line has particularities different from endogenous microglia in their native environment, the present results still pave the way to better appreciate the purinergic system in microglial cells as a putative target to be further considered for the management of glaucoma.

## Author contributions

AR-N, IA, and AS conceived and designed the experiments; AR-N, IA, JV, and FG performed the experiments; AR-N, IA, JV, RB, MM, FG, RC, PS, AA, and AS analyzed and interpreted the results; RC, PS, AA, and AS contributed with reagents, materials, analysis tools; AR-N wrote the first draft of the manuscript and all authors have read and approved the final version.

### Conflict of interest statement

The authors declare that the research was conducted in the absence of any commercial or financial relationships that could be construed as a potential conflict of interest.

## Abbreviations

A_1_R: adenosine A_1_ receptor
A_2A_R: adenosine A_2A_ receptor
A_2B_R: adenosine A_2B_ receptor
A_3_R: adenosine A_3_ receptor
ADA: adenosine deaminase
ADK: adenosine kinase
ADP: adenosine di-phosphate
AMP: adenosine monophosphate
ATP: adenosine triphosphate
BCA: bicinchoninic acid
BSA: bovine serum albumin
CD73: ecto-5′-nucleotidase
CNS: central nervous system
CNT: concentrative nucleoside transporter
ECF: chemi-fluorescence
ECL: Chemiluminescence
EHP: elevated hydrostatic pressure
ENT: equilibrative nucleoside transporter
E-NTPDase1/CD39: ecto-nucleoside triphosphate diphosphohydrolase 1
FBS: fetal bovine serum
HPLC: high-performance liquid chromatography
IOP: intraocular pressure
NBMPR: S-(4-nitrobenzyl)-6-thioinosine
PFA: paraformaldehyde
RGCs: retinal ganglion cells
RPMI: Roswell Park Memorial Institute
SDS: sodium dodecyl sulfate
TRITC: tetramethylrhodamine B isothiocyanate
VNUT: nucleotide transporter.
